# Relationship quality perceived by family caregivers of people with dementia in the context of a psychoeducational intervention: A qualitative exploration

**DOI:** 10.1177/14713012241264611

**Published:** 2024-08-06

**Authors:** Stephanie Kipfer, Cedric Mabire, Jean Vézina, Andrea Koppitz, Sandrine Pihet

**Affiliations:** School of Health Sciences Fribourg, 111832 HES-SO University of Applied Sciences and Arts Western Switzerland, Switzerland; Institute of Higher Education and Research in Healthcare-IUFRS, University of Lausanne, Lausanne University Hospital, Switzerland; Institute of Higher Education and Research in Healthcare-IUFRS, University of Lausanne, Lausanne University Hospital, Switzerland; School of psychology, 4440 University Laval, Québec, Canada; School of Health Sciences Fribourg, 111832 HES-SO University of Applied Sciences and Arts Western Switzerland, Switzerland

**Keywords:** dementia, family caregivers, dementia care, long-term care, relationship quality, psychoeducational intervention, longitudinal qualitative research, constructivist grounded theory, nursing

## Abstract

**Background:**

Caring for a person with dementia can be a challenging experience, often associated with chronic stress and a heavy burden on family caregivers. Dementia also impacts the relationship between the caregiver and the person with dementia. The quality of this relationship is, in turn, an important factor influencing the well-being of both dyad members. The psychoeducational intervention “Learning to feel better . . . and help better” has shown positive results regarding family caregivers’ subjective burden, psychological distress, and self-efficacy. However, relationship quality has not been addressed in the context of this intervention.

**Methods:**

A longitudinal constructivist grounded theory approach was used to explore relationship quality as perceived by caregivers, possible changes and intervention components facilitating or preventing such changes. Three qualitative, semi-structured interviews (before, during and after the intervention) were performed with 13 family caregivers from three different intervention groups. The resulting 39 interviews were analysed regarding individual caregiver trajectories, per time point for all caregivers and regarding specific caregiver subgroups.

**Findings:**

A model focusing on sustaining relationship quality in dementia was developed. It shows strategies that family caregivers develop and apply to facilitate positive interactions and feelings of connectedness with their family members with dementia. It also indicates that mastering such strategies requires reflective skills based on specific knowledge of dementia and coping strategies, which can be enhanced through active skills training, in which caregivers are guided to work on their individual stressful situations. Factors hampering change included difficulties in accepting dementia-related changes.

**Conclusion:**

Findings suggest that psychoeducation, with active skills training based on caregivers’ current daily life situations, providing systematic procedures to handle daily challenges and specific knowledge about the impact of the disease, could support them in developing and applying supportive strategies to sustain or improve their relationship to their family member with dementia.

## Introduction

Dementia is one of the main causes of disability and dependency in older people worldwide (World Health Organization, 2017), and the number of people affected is continually rising (Alzheimer’s Disease International, 2024; World Health Organization, 2017). Most people with dementia live in their homes (Wimo et al., 2018). As dementia progresses, the ability to manage one’s daily life independently becomes increasingly impaired, and people living with dementia become increasingly dependent on the support of others. This support is mostly provided by informal caregivers, who are predominantly female and spouses or adult children (hereafter referred to as family caregivers) (Alzheimer’s Association, 2023; Wimo et al., 2018). Family caregivers complete extensive and often complex caregiving tasks, including assistance and supervision with activities of daily living, emotional support, support with medication and treatment, symptom management, organisation and coordination of support services, as well as handling administrative and financial issues (Alzheimer’s Association, 2023).

Besides positive caregiving aspects, such as personal growth, gratification and strengthened relationships (Lindeza et al., 2020; Yu et al., 2018), caring for a person living with dementia can be a challenging experience (Lindeza et al., 2020). The cognitive and neuropsychiatric symptoms of the person with dementia, the unpredictable course of the disease and the straining and continuous care can cause high levels of stress and lead to physical, psychological, emotional, social and financial problems among family caregivers (Brodaty & Donkin, 2009; Gilsenan et al., 2023; Lindeza et al., 2020; Thompson & Roger, 2014). This phenomenon is known as the “caregiver burden” (Zarit et al., 1980). Poor health, in turn, impairs the quality of life of family caregivers and affects their ability to provide care and sustain their own social support network, leading to social isolation (Brodaty & Donkin, 2009; Lindeza et al., 2020; Thompson & Roger, 2014).

### The essential role of relationship quality

A growing body of research describes how the relationship between a person with dementia and their family caregiver may change over the course of dementia (e.g., Ablitt et al., 2009; Hochgraeber et al., 2023; Marques et al., 2024; Wadham et al., 2016). Caregivers often experience declines in the quality of their relationship to the person with dementia over time, as found in two recent longitudinal studies (Bruinsma et al., 2020; Marques et al., 2022). In general, relationship quality refers to “how positive or how negative individuals feel about their relationship” (Morry et al., 2010, p. 372). Declines reported by caregivers relate to mutual support, reciprocity, intimacy, affection, empathy, communication, possibility for shared activities, companionship and relationship happiness (Ablitt et al., 2009; Hochgraeber et al., 2023; Holdsworth & McCabe, 2018; Quinn et al., 2015; Wadham et al., 2016). For example, changes in the person with dementia’s behaviour or disease-related communication problems may complicate interactions within the dyad and negatively affect relationship quality (Conway et al., 2018; Stedje et al., 2023). Furthermore, shifts in roles, responsibilities and power within a family or dyad due to the reduced capabilities of the person with dementia may provoke conflicting emotions in the people involved or conflicts between them (Hochgraeber et al., 2023; Holdsworth & McCabe, 2018; Köhler et al., 2022; Wadham et al., 2016). Nevertheless, dyads endeavour to maintain their relationships, and positive aspects such as love, closeness, mutuality, emotional warmth and affection may remain intact or even increase during the experience of living with dementia (Ablitt et al., 2009; Hochgraeber et al., 2023; Holdsworth & McCabe, 2018; Marques et al., 2024; Wadham et al., 2016).

Research suggests that the quality of the relationship between a person with dementia and their family caregiver is an important factor related to the well-being and quality of life of both members of the dyad (Ablitt et al., 2009; Martyr et al., 2018; O’Shea et al., 2020; Quinn et al., 2009; Rippon et al., 2019). For example, relationship quality was a significant predictor of self-rated and proxy-rated quality of life in a European prospective cohort study conducted over a 12-month period (O’Shea et al., 2020). Relationship quality is also key to creating and maintaining a care situation at home (Köhler et al., 2021, 2022). In fact, a close relationship can be a strong motivator for family members to take on a caregiver role and can promote continuation of home care arrangements (Köhler et al., 2021, 2022). In particular, a continued sense of mutuality may act as a protective factor regarding the institutionalisation of a person with dementia (Hirschfeld, 2003; Marques et al., 2024).

Relationship quality may be influenced by multiple factors, as shown in a recent narrative synthesis systematic review focusing on spousal relationship quality (Stedje et al., 2023), which described 20 factors grouped into six categories: attitudes and strategies; behaviour and activities; emotional connectedness; activities and experiences outside the home; social behaviours and roles; and belonging and safety. Important factors include relationship quality before the onset of dementia (Ablitt et al., 2009; Hochgraeber et al., 2023), behaviour and psychological symptoms of the person with dementia (Marques et al., 2022; Stedje et al., 2023), individual and dyadic responses to dementia and related changes (Conway et al., 2018; Köhler et al., 2022; Stedje et al., 2023), as well as social support (Marques et al., 2022, 2024; Stedje et al., 2023).

### The need for effective support interventions

Faced with a complex caring situation over a long period of time, caregivers need to develop specific skills to successfully perform their role and manage the care situation while minimising negative outcomes. This includes taking on new responsibilities and coping with functional losses and behavioural changes, as well as changes in their relationship to the person with dementia (Bressan et al., 2020; Ducharme et al., 2011; Hochgraeber et al., 2023; Quinn et al., 2008). However, caregivers often have no experience in providing care, feel unprepared and may need support to develop the required skillset (Bressan et al., 2020; Reinhard et al., 2008).

There is increasing evidence that psychosocial interventions for caregivers are associated with small to moderate improvements in a range of caregiver outcomes, including caregiver subjective well-being, burden, depression, anxiety, general health, ability and knowledge, as well as in the symptoms of the person with dementia (Teahan et al., 2020; Walter & Pinquart, 2020). The most comprehensive meta-analysis to date, including 280 controlled studies on 332 interventions, indicates that psychoeducation with active skills training and multicomponent interventions showed the broadest effects, while other types of interventions only affected specific outcomes (Walter & Pinquart, 2020). The meta-analysis of Teahan et al. (2020), including 22 high-quality intervention studies, found a significant effect on caregiver burden for psychoeducational interventions but not for multicomponent ones, while both types of intervention had a significant impact on caregiver depression. Other relevant interventions focused on improving the relationship quality between the caregiver and the person with dementia. A recent scoping review of 34 such studies identified a broad range of interventions, ranging from common creative activities to psychotherapy (Gilbert et al., 2023). It, however, concluded that the identified benefits should be considered potential due to the limited methodological rigour of this body of evidence (Gilbert et al., 2023). Notably, both lines of research also point out important shortcomings. First, studies applied different designs, and interventions differed substantially regarding their type, content and mode of delivery (e.g., format, frequency or duration) (Laver et al., 2017; Teahan et al., 2020; Wiegelmann et al., 2021), which complicates their comparison and the identification of the most relevant features. Second, change mechanisms underlying positive results seem rarely examined, representing an important research gap, as such knowledge is essential to further develop and optimise an intervention (Cheng et al., 2019; Gilbert et al., 2023; Walter & Pinquart, 2020; Wiegelmann et al., 2021). Third, sociodemographic and clinical characteristics (e.g., age, type of dementia) require attention so that interventions can be tailored to the specific needs of caregiver subgroups (Cheng et al., 2019; Wiegelmann et al., 2021).

### Rationale and aim

This study focuses on the psychoeducational intervention: “Learning to feel better . . . and help better” (hereafter, the “Feeling better” intervention), a group intervention for family caregivers supporting a person with dementia living at home (Lévesque et al., 2002). “Feeling better” aims at empowering family caregivers by enhancing their skills to cope with the daily stress of dementia caregiving, including the management of dementia-related behaviours. In Canada, where the intervention was developed, a randomised controlled trial (RCT) showed a positive impact on the frequency of behaviour problems in persons with dementia and related distress in caregivers (Hébert et al., 2003). In Switzerland, this intervention has been shown to have a positive impact on caregivers’ subjective burden, psychological distress, self-efficacy and distress related to the behavioural problems of the person with dementia (Pihet et al., 2024; Pihet & Kipfer, 2018).

This intervention has undergone several stages regarding its development and evaluation in Switzerland (for an overview, see Pihet et al. (2024)). The qualitative analyses of the two foregoing feasibility and pilot studies (Pihet et al., 2024; Pihet & Kipfer, 2018) revealed important aspects to explore for a better understanding of how the intervention works. One such aspect was the relationship quality between the members of the caregiving dyad, as it likely contributed to and interacted with other changes reported by caregivers participating in the “Feeling better” intervention (Kipfer, Mabire, Vézina, et al., 2024; Pihet et al., 2024; Pihet & Kipfer, 2018). However, as improving relationship quality between the caregiver and the family member with dementia was not the intervention’s primary aim, this aspect was not explicitly explored or evaluated in the Swiss studies (Pihet et al., 2024; Pihet & Kipfer, 2018), nor in the intervention’s process evaluation in Canada (Lavoie et al., 2005). Given the relevance of relationship quality for the well-being and quality of life of caregivers and their family members with dementia, it is important to clarify whether and how this intervention may support caregivers in maintaining their relationship with the person with dementia. Knowledge of relationship quality and related mechanisms in the context of this intervention can provide insights to refine its theoretical underpinnings and improve the intervention itself. In addition, this knowledge is important for clinicians and researchers aiming to develop and provide effective interventions for dementia family caregivers or other interventions targeting similar populations or mechanisms (Kazdin, 2009; Moore et al., 2015).

Thus, the aim of this study was to qualitatively explore whether and how caregivers perceive changes in their relationships with the person with dementia in the context of the “Feeling better” intervention. More specifically, we aimed to explore: (1) changes occurring in relationship quality over the course of the intervention; (2) intervention components promoting such changes; and (3) contextual elements acting as facilitators or barriers regarding changes in relationship quality.

## Methods

### Design

A longitudinal, interpretative, constructivist grounded theory approach based on Charmaz (2006) was used to qualitatively explore the relationship quality perceived by family caregivers in the context of the “Feeling better” intervention. With their orientation towards symbolic interactionism, constructivist grounded theory approaches are particularly relevant for the in-depth exploration of social processes (Charmaz, 2006). Qualitative longitudinal studies allow us to explore how changes evolve over time, including related key time points, patterns, facilitators and hindrances (Fadyl et al., 2017; SmithBattle et al., 2018; Tuthill et al., 2020). This qualitative exploration of relationship quality was embedded in a larger study evaluating the “Feeling better” intervention.

### The intervention: “Learning to feel better . . . and help better” (“Feeling better”)

The Swiss version of the “Feeling better” intervention is provided to small groups of four to eight participants led by trained health professionals, such as nurses or psychologists. Based on Lazarus and Folkman’s transactional theory of stress and coping (Folkman et al., 1991; Lazarus & Folkman, 1984), caregivers learn a systematic procedure for assessing and coping with stressful situations (hereafter, the systematic procedure). They learn to break down a global situation into specific parts and distinguish between aspects or situations that can or cannot be changed. The intervention then addresses three coping strategies: (1) problem solving for modifiable situations; (2) reframing unhelpful thoughts for non-modifiable situations; and (3) support seeking for both types of situations, focusing on both formal and informal networks. Additional information is provided on dementia’s impact on communication and relational behaviour and how family caregivers can adapt their communication and prevent tensions. The Swiss version of the intervention consists of seven group sessions of 3 hours each (six weekly sessions plus a follow-up session one month later). It combines psychoeducation, including active skills training, to apply the systematic procedure and related coping strategies, with support group elements, including group work on personal stressful situations and roleplaying.

### Procedure

The intervention was delivered in German from September 2020 to June 2021 (i.e., during the second and third waves of COVID-19, thus with restricted group size) in the bilingual canton of Fribourg, Switzerland by a psychologist (SP) with four years of experience with the “Feeling better” intervention. Information on the intervention was published in local newspapers and distributed to professionals and organisations supporting family caregivers. Interested family caregivers contacted one of the course leaders and received detailed information about the intervention and the evaluation procedure. All caregivers willing to participate were invited to interviews about relationship quality, and their eligibility was assessed based on the following criteria: (1) regularly providing unpaid care to a person living at home and having a diagnosis of dementia or showing substantial cognitive deficits; (2) aged 18 years or older; and (3) having sufficient language skills in German. Written and oral informed consent was obtained from each participant before the start of data collection. The study was performed according to the Helsinki Declaration (The World Medical Association, 2022) and was approved by the local ethics review board of the Canton of Vaud, Switzerland (Protocol n°175/14; ISRCTN13512408).

### Data collection

Qualitative, semi-structured interviews were conducted with all participants (*N* = 13) in three consecutive intervention groups to collect data on perceptions of relationship quality and potential changes over the course of the intervention. All participants were interviewed at three time points: within 3 weeks before the beginning of the intervention (t0), after Session 5 (t1) to ensure the participants’ exposure to all core strategies of the intervention, and within three weeks after the end of the intervention (t2), resulting in a total of 39 interviews. The interviews were conducted by the main author according to the grounded theory approach of Charmaz (2006). The initial interview guide, with open-ended questions, was continuously adapted to gain complementary and in-depth knowledge. However, interviews at all time points started with the same two initial questions asking about their relationship with the partner or parent with dementia. Interviews at t1 and t2 also explored caregivers’ experiences since the last interview time point and aspects raised in their earlier interviews. This included exploring changes described by caregivers, related processes and intervention components (for interview guides, see Supplemental Material 1). Interviews were held at the university, at the participant’s home or by phone according to the caregiver’s preference and taking into account any COVID sanitary measures. Field notes summarised observations made during the interview and information about the interview context. Each interview lasted 34 minutes on average (range 11–74 minutes) and was audio-recorded, pseudonymized and verbally transcribed. The characteristics of the caregivers and their family members with dementia are described in Table 1.

**Table 1.** Characteristics of family caregivers and people living with dementia.

Characteristics of family caregivers (*n* = 13)		
Age^a^	Md = 66.00 years (63.50–71.50)	
Gender^b^	Female	*n* = 9 (69%)
	Male	*n* = 4 (31%)
Relationship type^b^	Spouse caregiver	*n* = 10 (77%)
	Child caregiver	*n* = 3 (23%)
Housing situation^b^	Living in same household	*n* = 10 spouse caregiver (77%)
	Living in a different household	*n* = 3 child caregiver (23%)
Duration providing care^a^	Md = 2.00 years (1.04 – 3.25)	
Time per week spent caregiving^a^	Md = 7.00 days (5.25 – 7.00)	
Characteristics of persons living with dementia (*n* = 13)		
Age^a^	Md = 76.00 years (68.50 – 84.50)	
Gender^b^	Female	*n* = 5 (38%)
	Male	*n* = 8 (62%)
Diagnosis^b^ (as reported by caregivers)	Alzheimer’s disease	*n* = 8 (62%)
	Frontotemporal dementia	*n* = 2 (15%)
	Dementia with Lewy bodies	*n* = 1 (8%)
	No specific dementia diagnosis	*n* = 2 (15%)
Time since diagnosis^a,c^	Md = 1.5 years (0.83 – 3.00)	
ADL (0–6)^a^	Md = 5.00 (4.25 – 6.00)	
IADL (0–8)^a^	Md = 4.00 (2.50 – 5.50)	

*Note.* ADL: Activities of daily living (Katz et al., 1970); IADL: Instrumental activities of daily living (Lawton & Brody, 1969); for ADL and IADL, higher scores indicate higher levels of autonomy.

^a^For metric or ordinal variables, descriptive statistics are median (Md), first and third quartiles (Q1 - Q3).

^b^For nominal variables, descriptive statistics are percentage and (frequency).

^c^*n* = 11.

Quantitative data collected from the same caregivers for the larger study, evaluating the “Feeling better” intervention, were used as additional data in the qualitative analysis. These data were used to analyse whether and how the qualitative data on the relationship quality of participants differed according to their results in the five quantitative outcomes. The aim was to gain broader insight into differences between caregivers and possible factors affecting the perception of, and changes in, relationship quality. The five outcomes collected before and after the intervention were caregiver’s burden, psychological distress and self-efficacy, as well as memory and behavioural problems in the person with dementia and caregivers’ associated distress (quantitative results published in Kipfer, Mabire, Vézina, et al., 2024).

### Data analysis

The qualitative data analysis started after the first interview and was subsequently performed in parallel with each new one (Charmaz, 2006). The first author coded the interviews in two steps: initial and focused coding. Codes were developed inductively; no predefined themes were used. Constant comparison was applied in all phases of the analysis to progressively develop more abstract constructs of the studied phenomena. Memos and informal analytic notes were continuously written to support the analysis and retrace the code and category development progress. Theoretical coding was used to clarify how focused codes are linked to each other in order to develop a theoretical model (Charmaz, 2006). Qualitative data were analysed (1) on an individual level, (2) per time point and (3) by comparing the data of specific subgroups. Analyses at the individual level (1) were performed to gain a deep and broad picture of whether, how, when and why relationship quality changed—or not—for each family caregiver. Regarding time points (2), similarities and differences were analysed in the data of all caregivers for each time point and overall. The qualitative findings of Steps 1 and 2 were then analysed regarding the differences and similarities between specific subgroups of caregivers (3) (e.g., between spouses and adult children). To further explore caregiver aspects potentially relevant in terms of perception and changes in relationship quality, we created groups based on the quantitative data and compared the qualitative data of caregivers who showed changes in quantitative outcomes (e.g., burden) with those who did not. Quantitative data were introduced into the qualitative analysis after analysing all qualitative data and timepoints of a complete intervention group, including the individual journeys of the respective participants, to avoid focusing on aspects that confirm the quantitative results (Moore et al., 2015). Matrices were created during analysis to compare the qualitative data (i.e., individual journeys, identified themes and processes) of different participants and subgroups (Miles et al., 2020). The ATLAS.ti 22 software aided the data analysis. A detailed description of the different analysis steps can be found in Kipfer, Mabire, Koppitz, and Pihet (2024).

Coding reliability was assessed for a random sample of excerpts coded by a second coder (SP) with the developed coding system (Polit & Beck, 2021). Differences were discussed and definitions refined accordingly. To further increase the reliability and credibility of the findings, the codes, their definitions and properties were regularly discussed with other researchers from different disciplines, focusing on the plausibility of interpretations to reduce interpretation errors (Polit & Beck, 2021). In addition, three health professionals (two course leaders of the French-speaking “Feeling better” intervention in Switzerland and one involved in the Canadian version) assessed the consistency of the preliminary findings with their experience in guiding and evaluating “Feeling better” groups. Finally, four family caregivers who participated in the interviews validated the findings regarding their experiences when participating in the “Feeling better” intervention (Charmaz, 2006).

## Findings

Based on the qualitative analysis, a model for sustaining relationship quality in dementia (hereafter, the relationship model) was developed. The model describes change processes in relationship quality from the perspective of caregivers. It shows strategies that family caregivers develop and apply to facilitate positive interactions and feelings of connectedness with their family members with dementia. It also indicates that mastering such strategies requires reflective skills based on specific knowledge of dementia and coping strategies. Such knowledge and skills may be fostered by active skills training, where caregivers are guided to work on their individual stressful situations and share knowledge and experiences with other caregivers. By illustrating how changes in relationship quality are enabled, the model focuses on supportive elements and processes in this regard. However, not all caregivers perceived changes in relationship quality at the same time point or to the same extent; a few perceived only subtle changes until the end of the intervention. Several contextual factors were identified as hampering caregivers’ learning processes and, thus, changes in relationship quality. These factors are conceptualised as barriers to change within the model.

**Figure 1.**
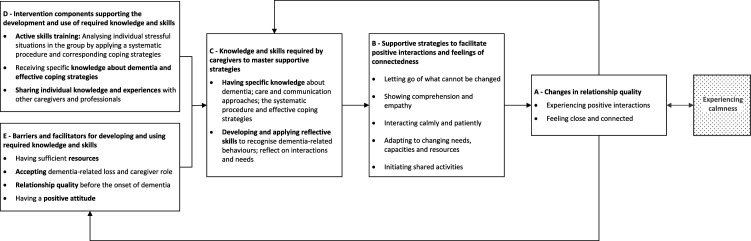
The model for sustaining relationship quality in dementia.

The relationship model (Figure 1) contains five components: A – changes in relationship quality; B – supportive strategies to facilitate positive interactions and feelings of connectedness; C – knowledge and skills required by caregivers to master supportive strategies; D – intervention components supporting the development and use of knowledge and skills; and E − barriers and facilitators for developing and using the required knowledge and skills. Figure 1 presents the relationship model with its five components, which are subsequently described from A to E in the findings below. When observed, differences between spouse and child caregiver are presented in the description of the respective components.

### A – Changes in relationship quality

Changes in relationship quality can be summarised in two themes: ‘Experiencing positive interactions’ and ‘Feeling close and connected’. These factors contribute to caregivers experiencing mutuality and reciprocity in their relationships with the person with dementia.

#### Experiencing positive interactions

Interviews at t0 were often characterised by narratives about changes in the behaviours of the persons with dementia (i.e., being stressed, agitated, accusing the caregiver, capacity loss and declining interest and lethargy) and stressful situations where the caregivers could not understand these behaviours.

“It happens quite often at the moment: when she’s stressed, when something doesn’t work, she starts to cry. Sometimes, I don’t know what to do in these situations”. (Caregiver (CG) 9/t1)

At t1 and increasingly at t2, most caregivers described situations where they experienced their parent or partner as calmer, showing less agitation, anxiety or distress. They further described the persons living with dementia as showing more motivation for shared activities, contributing more to daily life and willing to accept or ask for caregivers’ support. While some people with dementia regularly expressed gratitude for the support they received before the intervention, others started to do so over its course. The caregivers appreciated such gratitude and responded similarly. They reported that reducing inappropriate expectations and criticisms resulted in the person living with dementia no longer having to justify themselves, fight back or be stressed. In addition, better understanding and knowing how to address their relative’s needs helped them adapt activities accordingly and communicate with calm and empathy, which enabled positive interactions.

“Yes, indeed, we now have a good atmosphere. I even found my sense of humour again. […] Since I stopped correcting, criticising or blaming him, […] since I let all this go, he no longer blames me for things. It was like a reaction of his to my stressed behaviour. […] It was his way to defend himself. But he doesn’t have to do that anymore”. (CG2/t1)

In the post-intervention interviews, many caregivers reported a more relaxed atmosphere at home as conflicts decreased and positive interactions increased. They described more possibilities to share time in a pleasant way, laugh together and thus experience mutuality with the person with dementia.

“It does not feel the same anymore because the tension and stress caused by incomprehension are not here anymore. Yesterday, he could laugh heartily because I made a silly joke about something. This happened several times in the last fortnight”. (CG2/ t1)

#### Feeling close and connected

In the t0 interviews, several spouse caregivers reported experiencing or having actively taken some distance from their partners. They mostly described this distance as protection against painful emotions caused by the partner’s changed behaviours or losses related to the disease.

“It’s like a protective shield. It was not like this before. We can’t reach each other in the same way anymore. I am a person who suffers if I can’t reach the other person. I want to change this because [taking some distance] can’t be the solution. It does not make me happy”. (CG7/t1)

Feeling close and connected to their partner was increasingly mentioned in spouse caregiver interviews during and after the intervention. Several caregivers noticed having more positive feelings towards, and intimacy with, their partners. Some reported being less dismissive or kinder, and some felt connected again to their partner as a person rather than focusing on the difficulties associated with the disease. They attributed this increased closeness, affection and tenderness to their better understanding of, and responding to, their partners’ needs, leading to more positive interactions. They further attributed it to an improved understanding of their own feelings and thoughts and an increased awareness of their responsibilities and limits, helping them experience fewer negative feelings.

“I see our relationship in a different light. I can accept it better, and I behave differently towards him. I think I perceive him differently. […] Yes, he is still the person he was and my partner”. (CG3/t1)

The importance of physical closeness for creating proximity to their partner was mentioned by some spouse caregivers before the intervention. Others highlighted this during the intervention when realising that their partner was more receptive to emotional approaches than to cognitive inputs. They could then re-establish some closeness with their partners, showing more affection and tenderness, such as giving hugs, caressing their arm or massaging their neck. The latter showed that they appreciated these signs of affection and likewise took the initiative to exchange caresses. However, some caregivers reported having needs regarding physical affection that differed from those of their partners.

“Since I understand him better, I can also show more affection. And then sometimes, when he sits in his chair, I massage his neck, which he really enjoys. I was blocked; I wasn’t able to do it anymore. Now I can do it, and he is responding. […] He is my true mirror. When I give him calmness and caresses, he can give it back to me”. (CG2/t2)

All three child caregivers reported before the intervention that they felt closer to their parents since they took over a caregiving role, as they spent more time together and provided physical care. This closeness was maintained during and after the intervention or even increased when they could adapt their support strategies to prevent negative interactions. In addition, child caregivers improved their ability to perceive the needs and interests of their parents with dementia and engaged in representing them towards other people.

“She [sister of caregiver] didn’t understand; she felt he [parent with dementia] deliberately did not remember or listen. […] In these situations, I tell her: listen, it is not that he doesn’t want to remember. He just can’t”. (CG13/t2)

### B – Supportive strategies to facilitate positive interactions and feelings of connectedness

The qualitative analysis revealed five main supportive strategies (Figure 1, Component B) that facilitated the changes described above. Caregivers showed different patterns regarding which strategies they applied; not all of them applied all strategies. They also started to apply or reinforce supportive strategies at different time points during and after participating in the intervention.

#### Letting go of what cannot be changed

With this strategy, caregivers no longer invested efforts to change what they recognised as unmodifiable; instead, they modified their perceptions to become less stressed and less annoyed about unpleasant or difficult situations. This strategy included ceasing to criticise or correct the persons with dementia when they did things differently from expected, accepting their different experienced realities and not taking changed behaviours personally. This strategy helped caregivers adopt a more relaxed way of managing their everyday caregiving lives, which facilitated a calmer atmosphere and more positive dyadic interactions.

“His reality is the one that is true. This is a huge discovery for me; I cannot change it. I can handle this more relaxedly. To put it trivially: it is how it is”. (CG2/t2)

Another important element was recognising that they cannot control or take responsibility for everything their family members do or experience, nor always be at their family member’s side. This awareness was described as relief, and it sometimes enabled caregivers to give back some responsibility to persons with dementia, thereby supporting their autonomy. This, however, also included moments when the caregivers were uncertain if the persons with dementia would succeed in their intents, something they had to cope with. The strategy of ‘letting go of what cannot be changed’ also helped caregivers take things as they came, focusing more on the present and worrying less about the future. This attitude facilitated valuing and positively shaped the time spent with the family member with dementia.

“I realised that I cannot change anything there. I can try to make our time together as good as it can be without putting stress on myself. […] I think it is about realising that there are so many things that we cannot control. It is better to focus on the present”. (CG7/t2)

#### Showing comprehension and empathy

Caregivers were less irritated and responded with more comprehension and empathy when they could recognise changed behaviours as dementia-related and identify underlying needs. Some started to initiate conversations with the persons with dementia, addressing their negative emotions and worries by asking them about their needs and wishes, as well as by making them feel safe. Nonetheless, addressing negative emotions could also be very challenging and painful for caregivers, for example, when persons with dementia express grief about their decreasing abilities and worsening situations.

“In such [challenging] moments, showing affection instead of rejection and incomprehension: this was a core lesson for me, showing empathy, even in the most challenging situations. It had a profound effect on me, where before I was stuck with pure incomprehension”. (CG2/t2)

Most caregivers showed an improved understanding of the needs, perceptions and abilities of persons with dementia during and after the intervention. This helped them let their partner or parent do what made sense for them, even though they could not always fully understand it. It further led them to provide support in subtler ways to avoid confronting persons with dementia with their deficits and to use a positive attitude or humour to handle uncomfortable situations.

“I realise that I have to take over more and more tasks. I do this implicitly because I know he no longer knows how to do it. And if I correct him, I would probably provoke aggression”. (CG2/t1)

#### Interacting calmly and patiently

Before the intervention, many caregivers mentioned wanting to reduce their recurrent and painful conflicts with the person with dementia. During and after the intervention, caregivers reported trying to consciously adopt a calm and patient attitude when interacting with their partners or parents. They tried to calm down before responding and to remind themselves that the difficulties were caused by dementia and were not deliberate. This helped them to be gentler, giving calm answers (several times if necessary), avoiding pressure and giving their family member more time to respond. They were able to interact calmly in more situations and saw the benefits for the person with dementia and themselves. Nevertheless, staying calm in demanding situations was described as challenging because it required a high level of attention and training, which some caregivers were unable to maintain, particularly under time pressure or with other people around.

“With the course, I became aware that I needed to […] have more control over my emotions and no longer flare up from time to time. It still happens, but less. I first filter my emotions to better control them. I can see right away that this has a huge impact on him [partner with dementia] because he is a very anxious person”. (CG3/t2)

#### Adapting to changing needs, capacities and resources

During and after the intervention, most caregivers reported increasingly adapting to the changing needs, capacities and resources of both members of the dyad as they extended their knowledge about dementia and reflections about behaviours and interactions. This allowed them to be more aware of the present and declining capacities of the person with dementia and thereby to have appropriate expectations, which helped to reduce negative emotions for both of them.

“He is less stressed because I do not expect him to do things which he cannot fulfil. He is calmer. […] It almost seems like he is no longer afraid of my reaction. Somehow, now he can be how he is. This calms down the situation”. (CG2/t1)

Caregivers increasingly reported being able to recognise and reduce stressors, particularly by adapting activities for and with persons with dementia. This included allowing the person with dementia to do things independently by giving them enough time and avoiding pressure, maintaining routines and familiar procedures, creating a supportive environment, visiting familiar places and avoiding activities with a high risk of failure. Instead, caregivers tried to create activities related to the interests of the person with dementia, where they experienced stimulation and could use their own resources.

“He needs a lot of rest. It is no longer possible to do two things in one day. For example, if we go to a hairdresser, we cannot do shopping. Now I know this. […] Before, I usually told him, ‘Come on, you can do that. You just don’t want to’. This is no longer a topic. I no longer put pressure on him. I think this is a big change”. (CG2/t1)

#### Initiating shared activities

Shared activities were described at all time points as being important to feel connected, to maintain a shared identity and to sustain mutuality. Before the intervention, caregivers who still shared many interests and activities with their partner or parent with dementia described their relationship more positively than those who reported having lost most shared activities due to the disease.

“We have been married for a long time; we have a lot of shared interests. There is nature, hiking, reading, philosophising, and, yes, it still is a loving relationship”. (CG6/t0)

Caregivers mentioned initiating shared activities to make the person with dementia happy and to enable social contact and stimulation. However, they often reported difficulties adapting activities to the changing capacities of the person with dementia, while keeping them enriching for both members of the dyad, thereby preserving their bonding function. Most caregivers had already applied this strategy to some extent before the intervention. During and after the intervention, they showed an improved ability to adapt shared activities or develop new ones.

“We did handicraft together […]. This was a good moment. I was fascinated by how we were able to combine our skills […]. He had ideas I would never have thought of. […] And, of course, then I can compliment him”. (CG1/t2)

### C – Knowledge and skills required by caregivers to master supportive strategies

To develop and apply supportive strategies regarding relationship quality, caregivers used specific knowledge about dementia, care approaches and effective coping strategies, as well as reflective skills.

#### Having specific knowledge

The most important knowledge for caregivers was about the symptoms of dementia and its consequences on the affected person’s behaviour, as it helped them interpret the changed behaviour of their family member as dementia-related and not due to a bad attitude. This knowledge was important to understanding the reality of the affected persons and to having realistic expectations regarding their abilities, as well as to respond with more empathy and comprehension.

“Before, when he asked for the same thing the seventh time, I was like: ‘You are asking me again’, and I was a bit irritated. However, this is not the case at the moment. Now, I can tell myself, this is his disease, and his brain cannot process it. I have a greater understanding. Most of the time, I know why something is happening”. (CG1/t2)

Having knowledge about how to care for someone with dementia, particularly how to communicate, was mentioned by the caregivers as highly relevant. It helped them to better understand and support the person with dementia, for example by adapting activities or their own behaviour to the needs and capacities of the person. Other relevant knowledge was about supporting autonomy and stimulating activity in the person with dementia, which facilitated enriching and enjoyable shared moments.

Knowledge of the systematic procedure to appraise stressful situations and find effective coping strategies mainly helped caregivers guide their reflections on stressful situations in their daily lives. Most caregivers described the distinction between aspects that can or cannot be changed as particularly useful in deciding how to respond to a stressful situation and reduce negative emotions and interactions.

#### Developing and applying reflective skills

Reflecting on interactions and one’s own perceptions and needs, as well as on the needs of the family member with dementia, proved the foundation on which to build and apply supportive strategies. During and after participating in the intervention, caregivers provided more and more examples of how they increasingly applied reflective skills to improve their understanding, find solutions and manage the caring situation. Such reflection helped caregivers better understand how their family members perceived some situations and realise that some changed behaviours were triggered by simple needs that they could address.

“I felt kind of observed by him. […] I was so annoyed that he opened my door every morning to see if I was still there […]. Today, I know, he needs security […]. I can understand it. Therefore, it isn’t a topic anymore, while before I was quite annoyed”. (CG2/t1)

Reflecting on one’s own behaviours and perceptions involved observing how family members with dementia responded to specific behaviours or conditions. This helped caregivers gain relevant knowledge on supportive and stressing features, respectively, of how they could adapt their own behaviour or circumstances to avoid stressful interactions. Observing positive responses in their family members or themselves, in turn, helped them consolidate their knowledge and reflective skills, consequently supporting the related strategies, as indicated in Figure 1 by the arrow going from A to C. Through such key experiences, caregivers realised that they had the power to positively influence the situation of their family members with dementia. However, situations in which caregivers perceived contradictions between their needs and those of the affected person remained difficult to manage.

“[…] How does he [partner with dementia] respond when I do this? What do I have to consider with this person? What is important to me? How does he function? Every day, I learn and better understand what I have to do. What move do I have to perform with my bishop or my knight to avoid checkmating him? […] I am starting to get control over this game […]”. (CG2/t1)

### D – Intervention components supporting the development and use of knowledge and skills

Three intervention components were identified as helpful for caregivers to promote the knowledge and skills required to master supportive strategies. Receiving specific knowledge about dementia and effective coping strategies was important content, along with the delivery components of active skills training and the possibility to share individual knowledge and experiences in the group, which were essential for empowering caregivers in applying their new knowledge and skills in their everyday lives.

#### Active skills training in the group

where caregivers’ individual stressful situations were analysed with the help of the course leader and the active participation of the other caregivers, was found to be the most important component of the intervention. In each session, the course leader analysed several stressful situations experienced by different caregivers in their daily lives using the systematic procedure, including a relevant coping strategy. Stressful situations were often about challenging interactions with the person with dementia, and this systematic procedure trained caregivers to identify dementia-related behaviours, reflect on the needs of the affected person and find adapted solutions to enable positive interactions. It further prompted them to reflect on their own behaviours, emotions and needs. Back in their everyday lives, they tried to apply the solutions and reported their experiences in the following session, often resulting in successful moments or in the provision of further guidance in the case of (partial) failure. Roleplaying initiated by the course leader during this practical group exercise was described as challenging to perform but helpful in increasing their understanding regarding perceptions of the person with dementia or their dyadic interaction.

“There was a critical situation. He didn’t know where he was. We discussed this in the group. I found a solution when the situation happened, but in the course, I realised I could have managed it better. […] In the course, I understood that his reality did not match his current reality, so that I now can respond appropriately”. (CG5, t1)

#### Knowledge about dementia and effective coping strategies

were provided by the course leader during the group sessions, with the support of the course booklet and short educational videos, including testimonials of family dementia caregivers. This included knowledge about dementia and its consequences for the person affected, as well as communication and care approaches for persons with dementia. It further included guidance on how to reflect on stressful situations and how to choose and practically apply appropriate coping strategies (i.e., problem solving, reframing and support seeking). This knowledge was constantly applied when analysing stressful situations or performing roleplays during practical group exercises.

#### Sharing individual knowledge and experiences

Talking with other participants and the professional course leader about stressful situations allowed participants to share burdening experiences and feel understood by others, helping them normalise their emotions. Sharing enriching experiences and helpful strategies applied in daily life allowed them to acknowledge their skills. Moreover, it enabled them to learn from each other and thus further develop their repertoire of how to successfully manage stressful situations with their family members with dementia.

“The most positive thing for me was to talk, to tell other people what stresses me and then listen to their ideas about how to do it better”. (CG12/t2)

### E – Barriers and facilitators for developing and using required knowledge and skills

Caregivers differed regarding changes in relationship quality during and after the intervention. They showed different capacities to develop and apply new knowledge and skills and thus to develop and apply supportive strategies regarding relationship quality in their daily lives. Two main barriers were having limited resources and struggling to accept losses due to dementia or the caregiver role. Depending on the quality of the relationship before the onset of dementia, this could act as a hampering or facilitating aspect. Other facilitators were the ability to accept, to some extent, the disease and the caregiving role, as well as having a positive attitude, all three increasing caregivers’ readiness to develop and apply new knowledge and skills.

#### Having sufficient resources

Caregivers with limited physical, introspective or emotional resources experienced more difficulties in developing and applying new knowledge and skills. Limited physical or cognitive resources could be related to older age, a medical condition (e.g., heart problems) and/or exhaustion due to sleep deprivation.

#### Accepting dementia-related loss and caregiver role

Limited introspective or emotional resources were often connected to difficulties accepting the losses experienced or their caregiver role. This pattern occurred in some spouse caregivers who were oscillating between denial of and rebellion against their situation or were still stunned by the diagnosis, so they were not yet motivated to adapt to their new situation by developing new knowledge and skills. These caregivers often perceived the dementia-related behaviour changes as deliberate, causing them frequent and intense painful emotions, which further complicated the acceptance process. This pattern was particularly strong for two male spouse caregivers confronted with signs of frontotemporal dementia, who experienced a severe burden related to the particularly challenging behaviours of their spouses.

“It is a different person, 180 degrees the other way around. I can’t wrap my head around that. […]. We imagined things [our life] differently when I retired. I imagined things totally differently. Now, nothing happens anymore. We get up in the morning and go to sleep in the evening. It makes no sense anymore”. (CG10/t1)

Being able to see positive responses in the person with dementia or in themselves following the application of supportive strategies helped caregivers to reduce their negative emotions associated with dementia-related losses or their caregiver role, as indicated in Figure 1 by the arrow going from A to E. This supported them in moving on in their acceptance process.

#### Relationship quality before the onset of dementia

Taking on a caregiving role could also be complicated by the caregiver and the person with dementia having poor relationship quality before the onset of dementia. These few caregivers reported in their qualitative interviews having a very distant relationship with few interactions and a lack of emotional closeness due to negative dyadic experiences in the past. In this case, caregiving was often motivated by a sense of duty, causing ambivalent feelings about the caregiver role.

“I married because I had to. It wasn’t a love marriage. This makes it a lot more complicated, as I feel obliged to do this [taking over the caregiver role], but it does not really come from my heart. I can’t say he is my big love, and I will do everything for him, because I know if it were the opposite situation, he would probably not do it”. (CG12/t0)

#### Having a positive attitude

A positive attitude facilitated the learning and adaptation processes. Caregivers who already showed the ability to see the positive aspects of a situation, to focus on existing capacities rather than deficiencies, to use humour or to consciously enjoy pleasant moments, maintained or sometimes even intensified these during the course. They were further encouraged in this attitude when recognising positive responses in themselves or the person with dementia as a result of related strategies.

“[…] At the moment, it is still going well. Very often, we tell each other: ‘We still can have a lot of pleasant moments together’, and we want to enjoy this together”. (CG7/t0)

### Experiencing calmness and its link to relationship quality

As shown in Figure 1, caregivers reported another outcome related to relationship quality: experiencing calmness. Caregivers reported feeling less burdened, stressed, nervous or irritated in general, beyond simply in their interactions with their partner or parent with dementia. Some caregivers reported having changed from being constantly stressed in daily life to being stressed only in infrequently challenging situations. This perceived calmness was facilitated by other caregiver changes identified in the context of the intervention, such as having confidence in one’s own capacities, being able to act and to balance between caregiving demands and respite needs. Caregivers described a bidirectional link between experiencing calmness and relationship quality: experiencing this calmness, they reported being globally more patient, kind and understanding with their family member with dementia, which then facilitated positive interactions. Positive interactions, in turn, helped caregivers perceive their caregiving situation less negatively.

These findings led to the development of a second model: the calmness model. The calmness model focuses on caregivers’ strategies identified in the context of the “Feeling better” intervention and used in their daily lives to cope with stressful situations and experience more calmness (Kipfer, Mabire, Vézina, et al., 2024). By illustrating the overall change process in the context of this intervention, the calmness model conceptualises changes in relationship quality as one of five relevant intermediate changes interacting with calmness. Thus, the relationship model is embedded in the calmness model.

## Discussion

This study aimed to qualitatively explore the relationship quality perceived by caregivers participating in the “Feeling better” intervention. As a result, we developed a relationship model that describes supportive elements regarding relationship quality. These findings shall inform the intervention’s further development with a better understanding of the processes at work, which should improve the support for caregivers in achieving positive outcomes. By focusing on supportive elements, the model contributes to a positive perspective. Indeed, when developing support interventions in the context of dementia caregiving, it has been suggested to shift from ‘reducing stress’ to ‘optimising positive experiences’ (Yu et al., 2018, p. 24), as well as to focus on resource-oriented approaches to support couples (Bielsten & Hellström, 2019a).

### Observed changes in relationship quality

During and after the intervention, many caregivers reported situations in which they experienced more positivity in interactions with their family members with dementia and felt more connected to them. These findings indicate increased reciprocity and mutuality, respectively, and a better relationship quality perceived by caregivers. Feelings of closeness, maintaining a bond and enjoying time spent together are central to fostering a positive couple relationship in the context of dementia (Stedje et al., 2023). Feeling close was an important aspect related to relationship quality in our study, experienced by several spouse caregivers, especially when tenderness and affection were expressed. The importance of being affectionate and appreciative in sustaining couplehood in the context of dementia was also documented in a five-year longitudinal study (Hellström et al., 2007). Some spouse caregivers in our study highlighted the importance of physical closeness to their partners with dementia, as underlined by two reviews that found that redefining and continuing intimacy allowed for sustaining couplehood and fostering relationship quality in the context of dementia (Conway et al., 2018; Stedje et al., 2023). However, some of the caregivers in our study reported having different needs in this regard than their partners with dementia, which can be challenging for couples (Stedje et al., 2023). In their review, Conway et al. (2018) suggested that further research is needed to explore how couples living with this disease experience and conceptualise intimacy.

All three adult child caregivers reported feeling closer to their parents as a result of taking on the caregiver role and thus spending more time together, already before the intervention and even more in later interviews, due to their improved perception of their parents’ needs and more adapted strategies facilitating positive interactions. A longitudinal study including different types of caregivers (spouses, adult child, other relatives) found that some caregivers, who regularly interacted with their family member with dementia, felt emotionally closer to them at baseline and six-month interviews (Marques et al., 2024). At the one- and three-year follow-up, this closeness was impaired due to changed behaviours of the person with dementia and fewer shared activities or shifts in roles (Marques et al., 2024), suggesting that caregivers may need support to maintain their closeness to their family member as dementia progresses.

### Increased responsiveness as a core change fostering relationship quality

In our study, more positivity in interactions and increased feelings of closeness and connectedness were mainly facilitated by caregivers becoming more responsive to the person with dementia. Interview data indicated that the “Feeling better” intervention supported caregivers in better understanding the behaviours and needs of the people involved, as well as in adapting their own behaviour accordingly. The crucial role of responsiveness in close relationships is well known in the general population (e.g., Clark & Lemay, 2010). Understanding the perspectives, interests, desires and needs of the partner is essential to be responsive; responsiveness is, in turn, essential to feeling close and connected; and a responsive relationship is beneficial for the well-being of both partners (Clark & Lemay, 2010). In the context of dementia, responding in an empathic way by observing the current feelings and behaviour of the person with dementia and adapting their own behaviours accordingly was described as a supportive strategy regarding relationship quality (Köhler et al., 2022).

Caregivers reported less agitation, anxiety, distress or reluctant behaviour in their family members with dementia during and after the intervention, especially when caregivers could let go of criticising their partner or parent. This was mainly facilitated by caregivers being able to relate behaviours in their family members to dementia rather than to a bad attitude and thus to respond in a more understanding way. Taking behaviour less personally and adopting a more accepting, non-judgmental and non-blaming attitude have been described as beneficial in creating a solid base for relationships, particularly when couples have to cope with disability (Auclair et al., 2009). In Spruytte et al. (2002), a low level of conflict or criticism and a high degree of warmth were signs of relationship quality, whereas disturbances in the behaviour of the person with dementia and the caregiver’s perception that the latter had control over these behaviours were predictors of low relationship quality.

### Strategies identified as supportive to maintain relationship quality

Several of the strategies observed in this study as supportive of relationship quality are similar to those described in other research, mostly conducted outside of an intervention, and in particular, the study of Hellström et al. (2007). Based on 152 interviews conducted with 20 couples over five years in Sweden, Hellström et al. (2007, p. 405) described how couples in the context of dementia engage to sustain “a nurturative relational context”, that is “a milieu where their relationship can live and blossom”. In their study as well as in our study, caregivers tried to avoid stressors and provide subtle support to the affected person, with the aim of preventing family members with dementia from experiencing negative feelings. Hellström et al. (2007) described that caregivers tried to avoid triggers, increasingly took over tasks or hid caring activities in a subtle way to sustain the sense of reciprocity and maintain the involvement of the person with dementia. In the general population, the crucial element in providing support is to promote self-efficacy, whether the support is visible or invisible (Clark & Lemay, 2010). Caregivers in this study created conditions in which the person with dementia was able to actively take part in daily life, which is important for sustaining self-identity and couplehood (Conway et al., 2018; Hochgraeber et al., 2023). The strategy termed ‘Letting go of what cannot be changed’ consists, among other aspects, of focusing more on the present while valuing and positively shaping the time spent with the person with dementia. Similarly, two reviews mentioned taking one day after another as a coping strategy (Hochgraeber et al., 2023; Stedje et al., 2023). Hellström et al. (2007, p. 396) described “living for today” as an important strategy where couples give less thought to the future and focus on making “life as meaningful as possible while the opportunity existed”.

Maintaining activities and roles that are important for the dyad helps sustain the identity of both the caregiver and the person with dementia and allows them to connect with each other and feel part of a dyad (Conway et al., 2018; Hochgraeber et al., 2023). In our study, as in Hellström et al. (2007), couples initiated small, pleasant moments to sustain their couplehood. Child caregivers in our study often initiated activities in which the parent with dementia could spend time with the family, particularly with their grandchildren. Activities in which persons with dementia are socially engaged allow them and their family or friends to remain connected (Conway et al., 2018). Moreover, socialising as a dyad with other people outside the home and feeling accepted and appreciated as members of a group have been described as supportive of maintaining a positive relationship (Stedje et al., 2023).

Most strategies applied by caregivers in our study and identified as supportive of relationship quality relate to reframing, which was used as a form of emotion-focused coping, and to problem solving as a form of problem-focused coping (Folkman et al., 1991). As indicated by Lazarus and Folkman (1984), caregivers in our study often combined reframing (e.g., adapting expectations) and problem solving (e.g., adapting activities) to facilitate positive interactions. Other studies on the “Feeling better” intervention showed that reframing plays a key role for caregivers in managing the changed behaviours of the person with dementia, as well as their own negative emotions (Kipfer, Mabire, Vézina, et al., 2024; Lavoie et al., 2005; Pihet et al., 2024; Pihet & Kipfer, 2018). Further findings and discussion regarding the usefulness of coping strategies are provided in these publications.

### Empowering caregivers to apply knowledge and skills in their daily lives

Our relationship model highlights that the intervention’s change mechanism mainly relies on guiding caregivers in actively training their reflective skills and coping strategies, besides providing them with specific knowledge about dementia and coping strategies and the opportunity to share individual knowledge and experiences with other caregivers and professionals.

Specific knowledge of dementia and effective coping strategies are only useful when caregivers can transfer this knowledge to their situations and apply it in their daily lives. Our findings demonstrate the benefits of active skills training in this regard. Analysing participants’ stressful situations in the group with the active participation of the caregivers, using a systematic procedure including different coping strategies, facilitated such knowledge transfer and increased caregivers’ reflective skills. This fostered their understanding of their interactions with the person with dementia and thereby a more favourable attitude towards them. Well-developed reflection skills may help caregivers recognise positive responses or signs of affection that occur as subtle reactions during their interactions with their family members with dementia, thereby fostering relationship quality (Yu et al., 2018).

The importance of active skills training is supported by the results of an updated, comprehensive meta-analysis of interventions for dementia caregivers (Walter & Pinquart, 2020) suggesting that they should include active participation or training. Indeed, this meta-analysis showed that only skill-building psychoeducation with the active participation of caregivers (e.g., applying new theoretical knowledge to individual problems or performing roleplay) had positive effects on most outcomes, including caregiver burden, subjective well-being, depression or ability and knowledge. Interventions solely providing information, however, proved to impact caregivers’ abilities and knowledge only. A recent scoping review on strategies promoting the active participation of dementia family caregivers in psychoeducation (Chan et al., 2024, p. 14) cited the “Feeling better” intervention as incorporating three of the four core tenets of such strategies: “tailoring the content and formats to caregivers’ experiences, preferences and resources”; “facilitating peer sharing and support”; and “providing experiential learning opportunities” (i.e., roleplaying in our intervention).

Caregivers participating in the “Feeling better” intervention reported benefits such as feeling understood and less alone, having a safe place to talk about stressful experiences and emotions or learning and being encouraged by peers (Lavoie et al., 2005; Pihet & Kipfer, 2018). A review of psychosocial interventions described the group format as a key component in increasing the effectiveness of psychoeducational interventions (Dickinson et al., 2017). Experiences in terms of the group format and related social support processes are further addressed in other publications reporting on this intervention (Kipfer, Mabire, Vézina, et al., 2024; Lavoie et al., 2005; Pihet et al., 2024; Pihet & Kipfer, 2018).

### Including the dyadic perspective in “Feeling better”

Our findings suggest that the “Feeling better” intervention could support caregivers in increasing positive interactions and feelings of connectedness with their family member with dementia. Similar benefits could be achieved to some extent through a variety of psychosocial interventions for couples aiming at improving spousal relationships (Gilbert et al., 2023). A scoping review of 34 psychosocial interventions targeting dyads or their relationships, suggested that supporting dyadic communication and strengthening skills for coping with dementia-related changes in the relationship may contribute to enriching relationships (Hoel et al., 2023). Similar to this, while admittedly focusing on the caregivers, “Feeling better” provides specific knowledge and active skills training about dementia and associated communication and coping.

“Feeling better” could be further optimised by addressing relational aspects more explicitly. This includes providing knowledge on dementia-related changes in relationship quality and on supportive strategies (e.g., shared activities), as well as actively training their application during the intervention sessions and in caregivers’ daily lives as home exercises. This would be in line with the recommendation to include “an additional focus on the relational aspects of the caregiving dyad” to increase the effectiveness of interventions focusing on one member of the dyad (Hoel et al., 2023, p. 17).

Generally, the literature on relationships in dementia or on dyadic or couple interventions emphasises the importance of targeting both persons in the caregiving dyad simultaneously, considering both views and acknowledging them as a “unit interdependent on each other’s well-being” (Bielsten & Hellström, 2019a, p. 2447, 2019b; Hoel et al., 2023; Wadham et al., 2016). With its aim to improve caregivers’ coping skills, the “Feeling better” intervention focuses only on caregivers (Lévesque et al., 2002; Pihet et al., 2024). To better address the dyadic nature of relationship quality, the “Feeling better” intervention could be extended with dyadic modules, focusing, for example, on communication and shared activities. Such a combined format would allow a full dyadic approach for aspects where it is essential, such as active skills training focusing on the dyad (Van’t Leven et al., 2013) and the promotion of social inclusion (Bielsten & Hellström, 2019b). It would also preserve the benefits of the “caregivers-only” intervention, namely, providing caregivers with time dedicated to their own needs and an opportunity to freely express their feelings without fearing hurting the person with dementia. Indeed, “to succeed in continuing togetherness, not only the commonalities but also the separated activities of each member of the dyad are important to keep”, as they support the diverse identities of the persons involved and their common identities (Hochgraeber et al., 2023, p. 1971).

### Facilitators and barriers: Being ready to develop and apply new knowledge and skills

The caregivers in our study differed regarding the perceived changes in relationship quality. We found that an advanced age, limited physical or emotional resources and a distanced relationship, described by caregivers in the qualitative interviews before the intervention, were barriers to learning and applying new knowledge and skills, and in turn to maintaining or improving the relationship with the person with dementia. This finding is in line with the model of stress in dementia caregiving (Pearlin et al., 1990), where age, availability of personal resources and conflicts or distance in the past relationship can impact personal and social resources to deal with stress. The important role of prior relationships regarding current relationship quality has already been emphasised in previous research (e.g., Ablitt et al., 2009; Hochgraeber et al., 2023). Our findings indicate that a distanced relationship before dementia onset, as perceived by caregivers, can prevent them from serenely acknowledging their role and consequently from developing the knowledge and skills needed to improve their relationships. This is in line with Köhler et al. (2022), indicating that prior relationship quality plays an important role in how caregivers perceive and form their roles. Moreover, the quantitative study of Steadman et al. (2007) found that caregivers with less premorbid relationship satisfaction were likelier to react negatively to the behaviour of the person with dementia and had difficulties regarding communication and problem-solving skills. Such findings highlight that interventions aiming to support the relationship should consider prior relationship quality in order to set attainable goals and apply relevant approaches (Ablitt et al., 2009; Bielsten & Hellström, 2019a).

The most important barrier in our study was caregivers’ difficulties in acknowledging their situation, associated with an ambivalent state of denial coupled with strained negative emotions (e.g., strong anger, frustration and/or revolt). Perceiving a stressor as too threatening or uncontrollable can prompt disengaging coping strategies, such as denial or distancing (Wethington et al., 2015). Distancing was also observed in our sample, mainly before the intervention, as a strategy used by caregivers to protect themselves from experiencing painful emotions. Denial and distancing as forms of avoidance are maladaptive emotional regulation strategies associated with negative outcomes, such as psychological distress or subjective burden, and their use can prevent the adoption of healthier coping strategies (del-Pino-Casado et al., 2011; Gilhooly et al., 2016; Wethington et al., 2015). In contrast, the dementia grief model by Blandin and Pepin (2017) describes acknowledging loss and tolerating difficult emotions as core dynamic mechanisms that help caregivers move on in their grief process. As observed in our study, the authors described that acknowledging loss can be hindered when caregivers lack recognition of the loss, are resistant to or deny it, or are unable to acknowledge it emotionally. In turn, acceptance allows caregivers to actively choose and adopt a positive attitude, to be responsive and empathic with their family members with dementia and to adjust expectations and focus on possibilities rather than losses (Boots et al., 2015; Lloyd et al., 2016).

Having a positive attitude before the intervention was also found to facilitate caregivers’ learning processes. Other studies described a positive attitude towards challenges as supportive in dementia caregiving (Köhler et al., 2022, p. 11) or mentioned a positive mindset as an important factor (Hochgraeber et al., 2023). The facilitating role of both a positive attitude and acceptance of the situation could be explained by a higher sense of coherence helping caregivers actively engage in, and thus benefit from, a psychoeducational intervention, such as “Feeling better”. Sense of coherence refers to “one’s ability to understand a particular situation and use available resources effectively” (del-Pino-Casado et al., 2019, p. 14), based on global confidence that life is comprehensible, manageable and meaningful (Antonovsky, 1993). Among family caregivers, a higher sense of coherence is associated with lower levels of subjective burden and better mental health outcomes (del-Pino-Casado et al., 2019), as well as with higher relationship quality (Marques et al., 2022). In turn, family caregivers with a lower sense of coherence are at higher risk for negative outcomes and may be less responsive to psychoeducational interventions like “Feeling better”, as these interventions require active involvement and/or a solution-oriented attitude. Further research is needed to identify relevant interventions for this vulnerable subgroup of caregivers, as well as how to best support caregivers with limited resources (e.g., advanced age or health problems) and with difficulties in their grief processes within psychoeducational interventions. Moreover, the identified barriers and facilitators circumscribe caregiver factors, calling for further research exploring factors related to the intervention itself, such as the skills of course leaders delivering the intervention (Kipfer, Mabire, Vézina, et al., 2024).

### Limitations, strengths and future research

The qualitative exploration of relationship quality was embedded in a larger study evaluating caregivers’ experiences when participating in the “Feeling better” intervention. This allowed us to explore relationship quality from the perspective of the participating caregivers. However, relationship quality is a dyadic process, and one of the main limitations is, thus, the missing perspective of persons with dementia. Further studies must include the perspectives of both members of the dyad to better understand the interactions between them, as well as how people with dementia perceive their relationship to their partner or child caregiver (Gallagher-Thompson et al., 2020; Quinn et al., 2009; Rippon et al., 2019), for example by performing individual and joint interviews (Marques et al., 2024; Taylor & de Vocht, 2011).

Most participants were female and were spouse caregivers. Therefore, further research is needed to gain a deeper understanding of changes in relationship quality from the perspective of children caregivers and male caregivers. Information regarding the stage of dementia is limited to the diagnosis and the IADL and ADL scores reported by caregivers. As the study included only caregivers, no data could be collected from persons living with dementia. The study was conducted during the second and third waves of COVID-19, with different sanitary measures in force. Exploring their impact on caregivers or the care situation was not the aim of this study. As the sanitary measures were likely to impact contextual factors, such as caregivers’ social contacts or support possibilities, this aspect needs to be considered when interpreting the findings.

This is an uncontrolled study based on a small sample of caregivers. Therefore, findings regarding the benefits of the intervention need to be considered with due caution. However, the qualitative exploration and the developed model provide valuable insights that can be further investigated in a follow-up study with a larger sample.

The main strength of the current study was the longitudinal approach, which allowed us to explore relationship quality and changes over time, respectively, before, during and after the “Feeling better” intervention, thus providing rich and accurate data (Flick, 2019; Henwood & Shirani, 2022). Prolonged engagement with each participant enabled us to build a trusting relationship and talk about intimate themes. Furthermore, the longitudinal approach conducted with three different intervention groups at different time points and the different units of analysis enabled data triangulation. This further validated the data through multiple perspectives on the phenomenon, extended the knowledge about the phenomenon and allowed the development of a more comprehensive model (Flick, 2019; Polit & Beck, 2021). Including quantitative data in the qualitative data analysis allowed us to gain a more comprehensive view of the phenomenon (Flick, 2019). We found that comparing the qualitative data of caregivers with improved quantitative, post-intervention outcomes to those of caregivers showing stable or decreased outcomes was particularly helpful in exploring caregiver factors that facilitate or hamper change. Since it was unclear whether and how relationship quality may change in the context of the intervention, we first explored this with a qualitative design. However, adding a quantitative measure of relationship quality could have allowed the triangulation of quantitative and qualitative data on relationship quality. Nevertheless, our findings can inform future studies on how best to capture changes in relationship quality in the context of this intervention or similar ones.

## Conclusion

Empowering family caregivers to restore or maintain the quality of their relationship with their partner or parent living with dementia is a core public health issue, as it fosters the well-being of both dyad members and helps sustain the caring situation at home. Our model of sustaining relationship quality in dementia provides relevant knowledge for families affected by dementia and for health professionals, showing how caregivers can experience positive interactions and stay close to their family members with dementia, as well as how professionals could support them in this regard. The findings suggest that psychoeducational interventions with active skills training based on caregivers’ current daily life situations and providing systematic procedures to handle such challenges, as done in “Feeling better”, could support caregivers in developing and applying supportive strategies to sustain or improve their relationships with their family member with dementia.

## Supplemental Material

Supplemental Material - Relationship quality perceived by family caregivers of people with dementia in the context of a psychoeducational intervention: A qualitative exploration Supplemental Material for Relationship quality perceived by family caregivers of people with dementia in the context of a psychoeducational intervention: A qualitative exploration by Stephanie Kipfer, Cedric Mabire, Jean Vézina, Andrea Koppitz and Sandrine Pihet in Dementia

## Abbreviations

ADL: Activities of daily living
IADL: Instrumental activities of daily living
CG: Caregiver
RCT: Randomised controlled trial
