# Supplementary material for: Relationship quality perceived by family caregivers of people with dementia in the context of a psychoeducational intervention: A qualitative exploration
Source: Dementia (London). 2024 Aug 6;23(8):1263–91. doi: 10.1177/14713012241264611 (PMC11475760; doi:10.1177/14713012241264611)
Supplement: Supplemental Material - Relationship quality perceived by family caregivers of people with dementia in the context of a psychoeducational intervention: A qualitative exploration [file sj-pdf-1-dem-10.1177_14713012241264611.pdf]

Supplemental Material 1 – Qualitative Interview Guides

Interview Guide Relationship Quality – Pre-intervention Interviews

Please note, in line with the grounded theory approach applied in the study, that questions could be adapted or added in relation to the interview content, to deeper explore themes and related processes raised by the participants.

**Initial open-ended questions**
How would you describe your **relationship** with your partner/parent?<br><br>How do you feel about **your situation and that of your partner/parent** at the moment?<br><br>*Allow participants sufficient space and time to respond to the initial open-ended questions.*
**Examples of follow-up questions to deeper explore themes raised by participants**
Would you like to tell me more about the **changes in the relationship** with your partner/parent that you have described?<br>• How do you experience the **changes in the relationship** with your partner/parent that you have described?<br>• How do you feel about the **changes in the relationship** with your partner/parent that you have described?<br>• **When** did you notice these changes that you have described?<br>• What, do you think, **contributed to** this change you just described?<br>• How do you **deal** with these changes that you have described, in your **everyday life**?<br><br>Could you tell me more about the **changes in your partner/parent** that you have described?<br>• How do you experience the **changes in your partner/parent** that you have described?<br>• How do you feel about the **changes in your partner/parent** that you have described?<br>• **When** did you notice these changes that you have described?<br>• What, do you think, **contributed to** this change you just described?<br>• How do you **deal** with these changes that you have described, in your **everyday life**?<br><br>How would you describe the **atmosphere** in your relationship with your partner/parent?<br><br>How do you feel when you **spend time** with your partner/parent?<br><br>Could you tell me more about how you **spend time** together?<br>• How often do you do **activities together?**<br>• How do you experience these activities?<br><br>How **close** do you feel to your partner/parent?<br>• Is there any **affectionate touching** in your daily relationship, like hugging your partner/parent

or touching their shoulder? How do you experience this?

How would you describe the **communication** between you and your partner/parent?

- Do you **talk about your feelings** with your partner/parent, and does your partner/parent speak about theirs? How do you experience this?

How do you think your partner/parent **experiences the support** you provide?

How do you feel about the **support you provide** to your partner/parent?

- Often, when a person becomes ill, the **roles** in a relationship change. How do you perceive this issue with regard to your relationship with your partner/parent?

Final questions

Is there anything else I need to know to help me better understand your experiences?

Would you like to add something?

Interview Guide Relationship Quality – Interviews during and after the intervention

Please note, in line with the grounded theory approach applied in the study, that questions could be adapted or added in relation to interview content from current or previous interviews, to deeper explore themes and related processes raised by the participants.

**Initial open-ended questions**
How would you describe your **relationship** with your partner/parent?<br>How do you feel about **your situation and that of your partner/parent** at the moment?<br>*Allow participants sufficient space and time to respond to the initial open-ended questions.*
Examples of themes and follow-up questions *(based on the pre-intervention interview guide and the content of current and previous interviews with the same or different participants)*
• Perceived **atmosphere** in relationship with partner/parent, e.g.<br>*How would you describe the atmosphere in your relationship with your partner/parent at the moment?*<br>• Spending **time together**, e.g.<br>*How did you experience the activities that you undertook together?*<br>• Perceived **closeness** with partner/parent, e.g.<br>*How close do you currently feel to your partner/parent?*<br>• **Communication** within the dyad, e.g.<br>*How would you describe communication between you and your partner/parent at the moment?*<br>• Experiencing and dealing with partner's/parent's **changed behaviours**, e.g.<br>*How do you experience these behaviours? (refer to behaviour described by participant)*<br>• Perception of the **partner's/parent's response** to the **caregiver's support**, e.g.<br>*How do you think your partner/parent experiences the support you provide?*<br>• Feelings towards **support provided** to the partner/parent, e.g.<br>*How do you currently feel about your role within your relationship with your partner/parent?*
Examples of follow-up questions exploring changes and related processes described by participants
• How did you **notice the change**(s) that you have described? (refer to the change(s) described by the participant)

• How do you **feel about the change**(s) that you have described? (refer to the change(s) described by the participant)<br>• What, do you think, helped you to **handle the situation** in this way?<br>• Could you tell me more about what helped you **to apply this strategy**? (refer to the strategy/action(s) described by the participant)<br>• Could you tell me more about what you particularly **appreciated** about this/these **aspect**(s)? (refer to event/aspect raised by the participant)<br>• Could you tell me more about what was **challenging** for you regarding this/these **aspect**(s)? (refer to event/aspect raised by the participant)
**Final questions**
Is there anything else I need to know to help me better understand your experiences?<br><br>Would you like to add something?
